# Supplementary material for: A Targeted Catalytic Nanobody (T-CAN) with Asparaginolytic Activity
Source: Cancers (Basel). 2021 Nov 11;13(22):5637. doi: 10.3390/cancers13225637 (PMC8616244; doi:10.3390/cancers13225637)
Supplement: Supplementary file 1 [file cancers-13-05637-s001.zip › cancers-1451901-supplementary.pdf]

Supplementary materials: A Targeted Catalytic Nanobody (T-CAN) with Asparaginolytic Activity

Maristella Maggi, Greta Pessino, Isabella Guardamagna, Leonardo Lonati, Cristina Pulimeno and Claudia Scotti

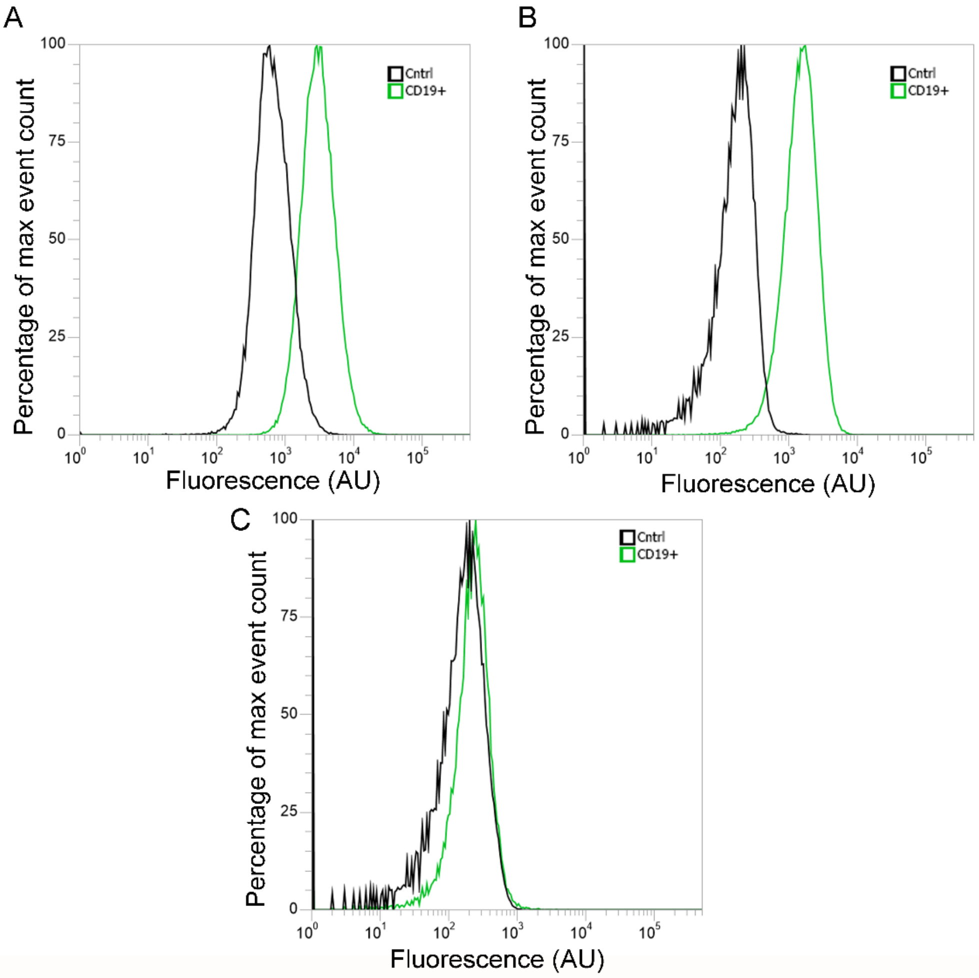

Figure S1. CD19 immunostaining. RAJI, REH and MOLT-4 cells were analyzed by flow cytometry for the expression levels of the surface marker CD19. In black: profile of control cells, in green:

profile of cells stained with an anti-CD19 antibody.

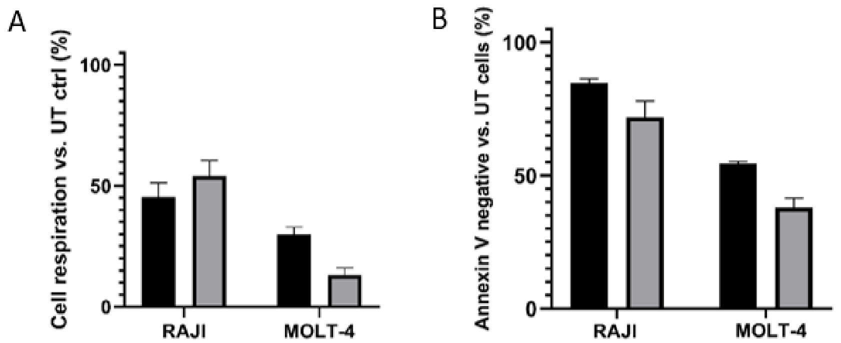

Figure S2. (A) and (B) MTT and Annexin V assays, respectively, performed on both RAJI and MOLT-4 cells with T-CAN (black bars) and Kidrolase (grey bars) at 0.15 U/ml. Values represents percentages versus untreated (UT) control.

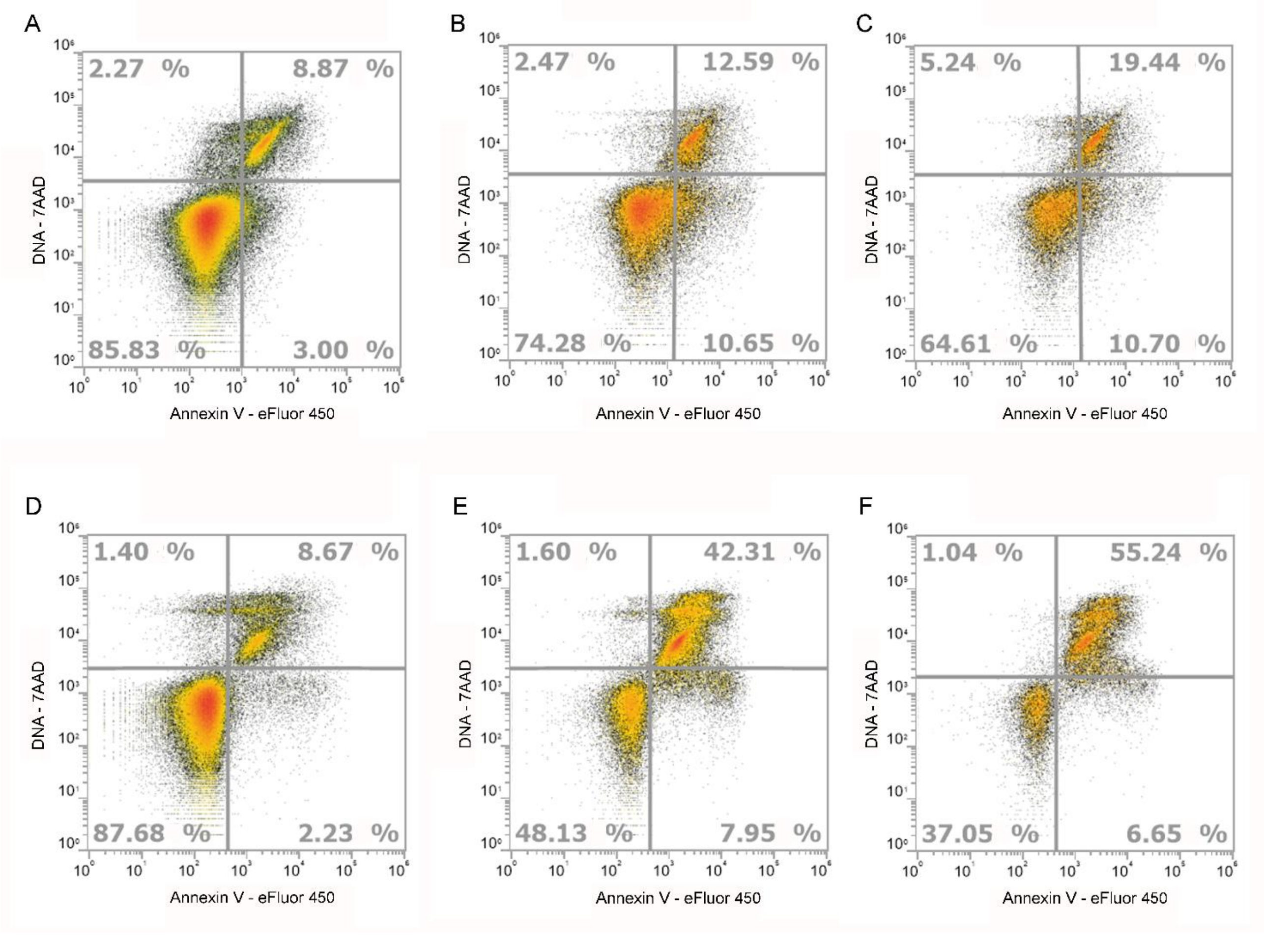

Figure S3. Annexin V/7AAD analysis representative FC plots. Panels A and D: RAJI and MOLT-4 untreated control (UT), respectively. Panels B and E: RAJI and MOLT-4 treated with 0.15 U/ml TCAN, respectively. Panels C and F: RAJI and MOLT-4 treated with 0.15 U/ml Kidrolase, respectively. Cells positive for 7AAD and Annexin v (bottom right, top left and right quadrants) were considered dead. Percentage of total events for

each quadrant are reported in grey.

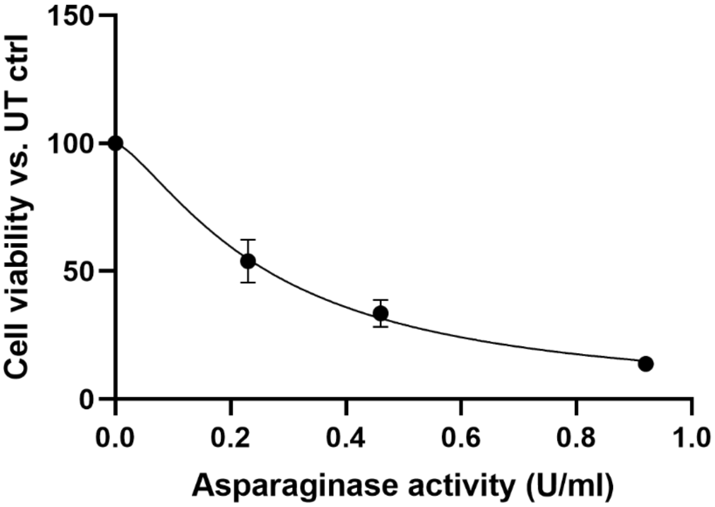

Figure S4. Viability of MOLT-4 cells treated with sdASNase, expressed as percentage versus untreated control (Trypan blue).

Blast IMGT database

VH gene

```
>20975 MK540646 Homo sapiens IGHV3-20*04 VH
          Length = 98

 Score =  120 bits (301), Expect = 2e-29
 Identities = 65/97 (67%), Positives = 77/97 (79%), Gaps = 3/97 (3%)

Query: 3  QVQLVESGGGTVPAGGSLRLSCAASTKTTDTYDMSWYRRAPGKGRDFVSGID-NDGTTTY 61
          +VQLVESGGG V  GGSLRLSCAAS  T D Y MSW R+APGKG ++VSGI+ N G+T Y
Sbjct: 1  EVQLVESGGGVVRPGGSLRLSCAASGFTFDDYGMSWVRQAPGKGLEWVSGINWNGGSTGY 60

Query: 62 VDSVAGRFTISQGNAKTA-YLQMDSLKPDNDTAMYYC 97
           DSV GRFTIS+ NAK + YLQM+SL+ + DTA+YYC
Sbjct: 61 ADSVKGRFTISRDNAKNSLYLQMNSLRAE-DTALYYC 96
```

Figure S5. Alignment of sdASNase to IMGT database best match for Homo sapiens V genes.

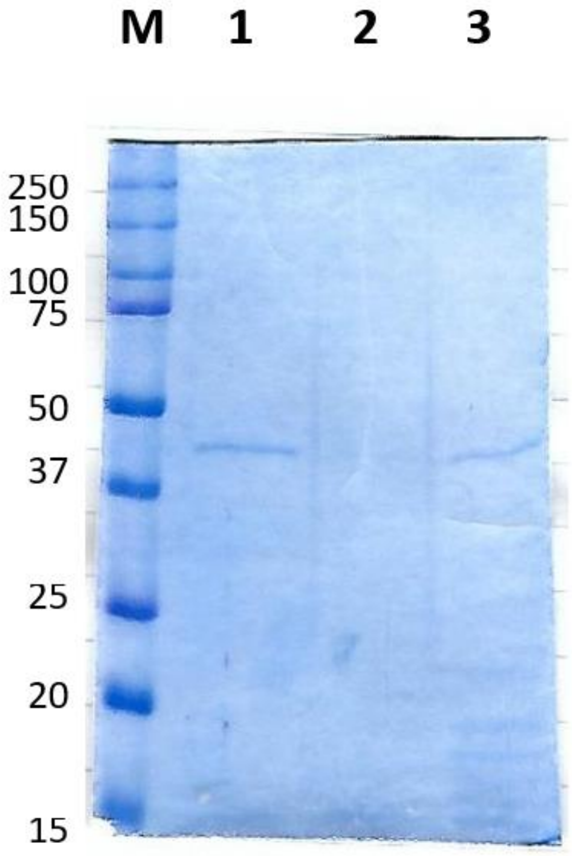

Figure S6. M: Marker (Precision Plus Protein standard, BioRad); lane 1: peak fraction at 250 mM imidazole elution, lane 2: flow-through; lane 3: preloading. Lane 2 and 3 were removed for the panel in Figure 4.
